# Adipokines, Metabolic Hormones and Their Associations with Abdominal Obesity against a Background of Hyper-LDL-C in Young People

**DOI:** 10.3390/jpm12111823

**Published:** 2022-11-02

**Authors:** Yuliya Ragino, Yana Polonskaya, Alexander Spiridonov, Evgeniia Striukova, Liliya Shcherbakova, Alena Khudiakova, Viktoriya Shramko, Ekaterina Stakhneva, Elena Kashtanova

**Affiliations:** Research Institute of Internal and Preventive Medicine—Branch of the Institute of Cytology and Genetics, Siberian Branch of Russian Academy of Sciences, 630089 Novosibirsk, Russia

**Keywords:** biochemical research in endocrinology, abdominal obesity, adipokine, leptin

## Abstract

Background: The present study was devoted to the search for possible associations between various adipokines/cytokines associated with the secretory activity of visceral adipocytes, elevated blood levels of LDL-C and abdominal obesity in people under 45 years. Methods: A population sample of Novosibirsk residents (n = 1415) was divided into deciles based on the levels of LDL-C. The study included 158 people, 87 men and 71 women, who had serum LDL-C levels of ≥4.2 mmol/L. Abdominal obesity was found in 50% of people (54% men, 45% women). By multiplex analysis using the human metabolic hormone V3 panel and the human adipokine magnetic bead panel, levels of adipokines and inflammatory markers were determined on a Luminex MAGPIX flow fluorimeter. Results: According to multivariate regression analysis (binary logistic regression), the most significant biomolecules, regardless of other factors, associated with the presence of AO against the background of hyper-LDL-C in young people were leptin (direct association) and lipocalin-2 (reverse association), leptin in young men (direct association), and leptin and TNF-alpha in women (direct association). Conclusions: Thus, in young people under 45 years with the presence of two important, potentially atherogenic risk factors—hyper-LDL-C and abdominal obesity—a complex of adipokines and metabolic hormones were associated with the presence of these diseases.

## 1. Introduction

In recent years, abdominal obesity (AO) has become more common. Data from many studies suggest that AO leads to chronic non-communicable diseases [1,2,3,4]. This indicates the importance of studying the association of AO with the pathogenesis of common therapeutic diseases and their risk factors [1,2]. Observations suggest that AO is an unfavorable form of obesity with serious consequences [5]. The European Society of Cardiology/European Atherosclerosis Society (ESC/EAS) guidelines give the definition of AO as the increased waist circumference indicating AO, regardless of the body mass index (BMI), is the waist circumference of ≥94 cm in men and ≥80 cm in women [6].

Adipose tissue, in addition to its energy storage function, acts as an endocrine organ that produces and secretes several biologically active substances, such as adipokines/cytokines [7]. AO is a risk factor for the development of cardiovascular diseases (CVD)because of chronic low-grade inflammation stimulated by adipokines/cytokines secretion by visceral adipocytes [8,9].

Elevated blood levels of low-density lipoprotein cholesterol (LDL-C) is one of the key risk factors for coronary heart disease (CHD) and atherosclerosis [6]. Currently, a very small number of studies have been conducted to examine the association between various adipokines (potentially atherogenic and potentially anti-atherogenic, pro-inflammatory and anti-inflammatory) with elevated levels of LDL-C in the blood [10,11,12,13,14,15]. Research data in this area often has contradictory results. It is also important to note that all these studies were conducted in patients over the age of 45, when the hormonal status of the body begins to change due to a decrease in the levels of sex hormones. It has been proven that in people older than 45 years, a decrease in the level of sex hormones is correlated with an increased in age-associated cardiometabolic risk [16,17]. In this regard, it seems relevant to study the features of the association between adipokines and cardiometabolic risk factors in a young population.

The relevance of adipokines/cytokines studies and their relationship with the traditional risk factors of cardiovascular diseases, in particular with elevated levels of LDL-C, is important since AO has a significant impact on the development of cardiovascular and many other noncommunicable therapeutic diseases. Adipokines and cytokines secreted by visceral adipocytes in abdominal obesity potentiate the development of insulin resistance, chronic inflammation and, as a result, hyperlipidemia, including hyper-LDL-C [18,19].

The present study was devoted to the investigating the possible association between different adipokines/cytokines with AO in young people under 45 years of age with elevated blood levels of LDL-C.

## 2. Materials and Methods

The study was designed as a single-stage population study of the Novosibirsk residents. The study was approved by the Local Ethics Committee (Protocol № 167 of 26.11.2019).

To build a population sample, the base of the Territorial Compulsory Health Insurance Fund for Persons aged 25–44 was used for one of the districts of Novosibirsk, typical in industrial, social, population-demographic, transport structures and the level of migration of the population. A random representative sample of 2500 people was formed using a random number generator. Young age groups are known to be among the most rigid to respond; therefore, methods of phased epidemiological stimulation were applied: mail invitations, phone calls and information messages in the media. At the screening, 1415 people were examined. Informed consent was obtained from all persons before the examination and processing of personal data.

### 2.1. Screening

A team of doctors trained in standardized epidemiological methods of screening examinations conducted the screening. The survey program included demographic and social data, a survey on the smoking habits and alcohol consumption, a socio-economic survey, a dietary survey, a history of chronic diseases and medication use, a Rose angina questionnaire, anthropometry, threefold measurement of blood pressure (BP), spirometry, and ECG recording with transcription according to the Minnesota code and others.

The waist circumference was determined with a tape in centimeters, applying it horizontally in the middle between the lower edge of the costal arch and the sacral part of the ilium. Abdominal obesity was established at a waist circumference in men of ≥94 cm, and in women of ≥80 cm [6].

Blood pressure was measured three times with an interval of 2 min on the right hand in a sitting position after a 5 min rest using an Omron M5-I automatic tonometer (OMRON Healthcare, Tokyo, Japan) with the registration of the average value of three measurements. Arterial hypertension (AH) was recorded at a systolic blood pressure (SBP) ≥ 140 mmHg and/or a diastolic blood pressure (DBP) ≥ 90 mmHg [6].

To assess insulin resistance, the HOMA-IR index was determined, which was calculated using the formula HOMA-IR = glucose concentration (mmol/L) × insulin level (µIU/mL)/22.5. The reference values of the HOMA-IR index were considered between 0–2.7.

### 2.2. Blood Tests

A single blood sampling from the ulnar vein was performed on an empty stomach after 12 h of fasting. Blood serum parameters of the lipid profile (total cholesterol (TCH), LDL-C, triglycerides (TG) and HDL-C) and glucose were measured by enzymatic methods using standard Thermo Scientific reagents (Thermo Fisher Scientific, Vantaa, Finland) on an automatic biochemical analyzer KoneLab 30i (Thermo Fisher Scientific, Vantaa, Finland).

The entire population sample was divided into deciles based on the levels of LDL-C. This study included 158 people, 87 men and 71 women from the last decile based on the levels of LDL-C in the blood. This level of LDL-C was recorded at ≥4.2 mmol/L. In accordance with the ESC/EAS guidelines 2019, the recommended value of LDL-C for people without severe diseases (low risk) should be less than 3.0 mmol/L [6].

### 2.3. Multiplex Analysis

By multiplex analysis on a Luminex MAGPIX flow fluorimeter (Austin, TX, USA), two panels were determined in blood serum:

(1) The human adipokine magnetic bead panel 1: adiponectin, adipsin, lipocalin-2/NGAL, PAI-1 (Total), resistin (EMD Millipore’s MILLIPLEX, Darmstadt, Germany);
(2) The human metabolic hormone V3 panel: amylin (total), C-peptide, ghrelin (active), glucose-dependent insulinotropic polypeptide (GIP), glucagon-like peptide-1 (GLP-1) (Total), glucagon, interleukin-6 (IL-6), insulin, leptin, monocyte chemoattractant protein-1 (MCP-1), pancreatic polypeptide (PP), polypeptide YY (PYY), tumor necrosis factor-α (TNFα), secretin (EMD Millipore’s MILLIPLEX, Darmstadt, Germany).

### 2.4. Statistical Analysis

Statistical processing was performed using the SPSS software package (version 13.0) (SPSS, Chicago, IL, USA). The data obtained in the tables and text are presented for categorical variables as absolute and relative values n (%), in the case of continuous variables as Me and (25%; 75%), where Me is the median, 25% and 75% are the 1st and 3rd quartiles, due to the abnormal distribution of most data (normality of distribution was checked by the Kolmogorov–Smirnov criterion). To assess the differences, we used a nonparametric criterion for comparing two independent samples, the Mann–Whitney U-test. The evaluation of variable associations was evaluated using multivariate logistic regression analysis, which was performed in compliance with the following conditions: dependent variable dichotomous (presence/absence of AO); independence of observations; absence of multi-collinearity, i.e., situations where the independent variables strongly correlate with each other (*r* > 0.9); linear dependence between each independent variable and the logarithm of the odds ratio (log_odds_); independence leftovers. The results of multiple logistic regression analysis are presented as OR and 95% CI for OR. At the first stage, an analysis was carried out with the inclusion of all the studied indicators; at the second stage, the results were confirmed by the results of a step-by-step (direct inclusion) logistic regression analysis. The critical significance level of the null statistical hypothesis (*p*) was assumed to be 0.05.

## 3. Results

This study included 158 people, 87 men and 71 women from the last decile based on the levels of LDL-C in the blood: recorded at ≥4.2 mmol/L.

Abdominal obesity (waist circumference in men of ≥94 cm, waist circumference in women of ≥80 cm) was detected in 79 people (50%), including 47 men (54%) and 32 women (45.1%).

We conducted a comparative analysis of clinical characteristics between individuals with hyper-LDL-C with AO and without AO. In persons with hyper-LDL-C and AO, the indicators of systolic blood pressure (SBP) were 1.1 times higher (128.3 ± 18.1 vs. 120.3 ± 13.8 mmHg), the indicators of diastolic blood pressure (DBP) were 1.1 times higher (85.9 ± 11.7 vs. 79.3 ± 9.4 mmHg), the prevalence of hypertension was 2.0 times higher (33.3% vs. 16.5%), the high level HOMA-IR index was 1.25 times higher (90.9% vs. 72.4%), the blood level of TGs was 1.4 times higher (1.8 ± 1.2 vs. 1.3 ± 0.6 mmol/L) and the blood level of HDL-C was 1.1 times higher (1.2 ± 0.3 vs. 1.35 ± 0.3 mmol/L), in comparison with persons with hyper-LDL-C without AO.

A similar analysis was carried out in men and women. In men with hyper-LDL-C and AO, the DBP was 1.1 times higher (89.0 ± 11.3 vs. 82.1 ± 9.4 mmHg) and the blood level of TGs was 1.5 times higher (2.1 ± 1.3 vs. 1.4 ± 0.6 mmol/L), compared with men with hyper-LDL-C without AO. In women with hyper-LDL-C and AO, the DBP was 1.1 times higher (81.2 ± 10.8 vs. 76.5 ± 8.6 mmHg) and the blood level of HDL-C was 1.15 times lower (1.3 ± 0.3 vs. 1.5 ± 0.3 mmol/L), compared with women with hyper-LDL-C without AO.

Further, we conducted a comparative analysis of blood levels of adipokines/cytokines associated with the secretory activity of visceral adipocytes among individuals with hyper-LDL-C with AO and without AO (Table 1). Individuals with hyper-LDL-C and AO had higher blood levels of C-peptide by 1.6 times, GLP-1 by 1.25 times, leptin by 2.6 times and TNF-α by 1.4 times, compared with individuals with hyper-LDL-C without AO.

A similar analysis was carried out in men (Table 2) and women (Table 3). Men with hyper-LDL-C and AO had higher blood levels of C-peptide by 1.9 times, leptin by 2.7 times and lower blood levels of lipocalin-2 (NGAL) by 1.7 times, compared with men with hyper-LDL-C without AO. In addition, men with hyper-LDL-C and AO had a tendency (*p* = 0.054) to have a higher blood level of glucagon-like peptide 1 (GLP-1) by 1.3 times, compared with men with hyper-LDL-C without AO. Women with hyper-LDL-C and AO had higher blood levels of C-peptide by 1.4 times, glucagon-like peptide 1 (GLP-1) by 1.15 times, leptin by 3.1 times and TNF-α by 1.8 times, compared with women with hyper-LDL-C without AO.

Further, we conducted a multivariate regression analysis (binary logistic regression analysis) of the association of adipokines/cytokines with the presence of hyper-LDL-C against the background of abdominal obesity. The presence/absence of AO against a background of hyper-LDL-C was included in the model as a dependent variable, and adipokines/cytokines (separately and in small groups), as well as age, gender, BMI, smoking status, blood pressure, blood lipids and other factors were included as covariates. In the general group, in a step-by-step regression analysis the chance of having AO against a background of hyper-LDL-C was, independent of other factors, directly associated with blood levels of leptin, TGs, DBP and being male and inversely associated with blood levels of lipocalin-2 (Table 4). In men, in a step-by-step regression analysis the chance of having AO against a background of hyper-LDL-C was, independent of other factors, directly associated with the levels of leptin in the blood and DBP (Table 5). In women, the chance of having AO against a background of hyper-LDL-C was, independent of other factors, directly associated with blood levels of leptin, TNF-α, DBP and TGs (Table 6).

## 4. Discussion

In our study, we obtained results showing higher blood levels of GLP-1 in persons under 45 years of age with hyper-LDL-C and AO, including in women, compared with persons with hyper-LDL-C without AO. Discussing our results, it should be noted that GLP-1 is an insulinotropic postprandial peptide hormone from the incretin family. The data from various studies on this hormone in obese people are very contradictory. For example, some studies have shown a decrease in the postprandial level of GLP-1 in obese individuals compared to those with a normal body weight. It has been demonstrated that the postprandial GLP-1 secretion is inversely proportional to BMI. In other studies, no differences in the levels of GLP-1 in the blood between obese and normal body weight were found [20]. There is evidence that GLP-1 is a synergist with adiponectin [21]. In any case, almost all studies, unlike ours, were conducted with the participation of obese patients over 45 years old, when in general the activity of the hormonal status of the body, especially in women, decreases.

Further, in our study, we revealed higher levels of TNF-α in the blood of persons un-der 45 years of age with hyper-LDL-C and AO, including in women, compared with persons with hyper-LDL-C without AO. In addition, we found that in women, the chance of having hyper-LDL-C and AO, regardless of other factors, is directly associated with the levels of TNF-α in the blood. The result obtained do not contradict the results of other studies. This cytokine, secreted by visceral adipocytes in AO, participates in the transmission of various cellular signals, and its excess is the cause of insulin resistance [22,23]. In obese people, including those with AO, the blood levels of TNF-α are increased [24,25].

In our study, we obtained results showing lower blood levels of lipocalin-2/NGAL in men under 45 years of age with hyper-LDL-C and AO, compared with men with hyper-LDL-C without AO. In addition, we found that in all people under 45 years the chance of having AO against a background of hyper-LDL-C, independent of other factors, was inversely associated with the levels of lipocalin-2 in the blood. Discussing this result, it should be noted that NGAL is synthesized by various cells, including visceral adipocytes, and NGAL is an adipocytokine and a component of the immune system, possessing antibacterial and anti-inflammatory properties. The levels of lipocalin-2 in tissues increases with metabolic disorders such as obesity and type 2 diabetes, indicating a link between lipocalin-2 and insulin sensitivity and glucose homeostasis. It has been reported that the blood levels of lipocalin-2 are higher in people with metabolic syndrome; however, there were only 18 patients with metabolic syndrome in the study [26]. The exact role of this adipocytokine in the modulation of insulin sensitivity, glucose and lipid metabolism is still unclear [27]. Thus, our result regarding the lower content of this adipocytokine in the blood of young men with AO and hyper-LDL-C is new, since this biomolecule has not been studied in men under 45 years of age with hyper-LDL-C and AO.

We found that people under 45 years of age with hyper-LDL-C and AO, both men and women, have higher blood levels of C-peptide. Our results do not contradict the data from other studies. The concentration of C-peptide in the blood reflects the level of insulin in the blood, since this biomolecule is secreted by beta cells of the pancreas when proinsulin is broken down to insulin. It has been shown that insulin potentiates a decrease in total cholesterol and LDL-C in the blood [28]. On the contrary, people with obesity and, especially, with AO, develop insulin resistance and the level of insulin and C-peptide in the blood increases. This leads to the activation of atherogenesis by several mechanisms, such as by stimulating lipogenesis, proliferation of vascular smooth muscle cells and activation of genes involved in the inflammatory process [29].

Finally, we obtained data on a significant, independent of other factors, direct association of blood leptin levels with the presence of hyper-LDL C and AO in young people under 45 years of age. Thus, people under 45 years of age with hyper-LDL-C and AO, both men and women, have higher blood levels of leptin compared to people with hyper-LDL-C without AO. In addition, according to multivariate regression analysis in people under 45 years of age, both men and women, the chance of having AO against a background of hyper- LDL-C, independent of other factors, is directly associated with the levels of leptin in the blood. This confirms the significant role of adipokine secreted by visceral adipocytes, especially in leptin resistance, in the development of potentially atherogenic changes in young people under 45 years. Our data do not contradict the data of numerous studies in patients over the age of 45 years. Thus, dyslipidemia with increased levels of total cholesterol and LDL-C has been observed in patients with defects in leptin function [30]. On the one hand, one of the mechanisms responsible for leptin resistance is inflammation; on the other hand, leptin has the properties of a pro-inflammatory cytokine and directly affects the inflammatory response by activating monocytes, leukocytes and macrophages to produce interleukins, cytokines and chemokines [31]. Hyperleptinemia is considered as an independent risk factor for CHD and is a significant prognostic factor for acute myocardial infarction [32]. It has been shown that leptin induces the expression of C-reactive protein, cell adhesion molecules and tissue thromboplastin in human coronary artery endothelial cells and induces oxidative stress and hypoxia. This contributes to endothelial dysfunction and indicates the proatherogenic role of leptin in hyperleptinemia [33,34].

## 5. Conclusions

In conclusion, it is important to emphasize that in young people up to 45 years with the presence of two important, potentially atherogenic risk factors—hyper-LDL-C and abdominal obesity—we examined a complex of adipokines and metabolic hormones associated with the secretory activity of adipocytes. In young people, leptin (direct association) and lipocalin-2 (reverse association), in young men leptin (direct association), in women leptin and TNF-α (direct associations) are the most significant biomolecules, regardless of other factors, associated with the presence of AO against a background of hyper-LDL-C. The obtained results reflect the important, perhaps main role of leptin in metabolic and lipid disorders in abdominal obesity in people under 45 years of age.

## 6. Study Limitation

The number of persons included in the study is small (158 people). The design of the study was to examine only individuals from the 10th decile of the distribution of LDL-C levels in the population aged 25–44 years.

## Figures and Tables

**Table 1:** Analysis of blood levels of adipokines/cytokines associated with the secretory activity of visceral adipocytes in people with hyper-LDL-C with and without AO (Me (25%; 75%)).

Parameters	LDL-C 10th Decile, AO (−) N = 79	LDL-C 10th Decile, AO (+) N = 79	*p*
Amylin, pg/mL	9.7 (7.4; 16.7)	12.8 (8.3; 19.9)	0.085
C-peptide, ng/mL	0.7 (0.5; 0.9)	1.1 (0.8; 1.8)	0.0001
Ghrelin, pg/mL	86.8 (29.2; 138.8)	77.3 (39.4; 123.4)	0.858
Glucose-dependent insulinotropic polypeptide, pg/mL	25.3 (16.8; 38.6)	25.3 (17.3; 48.2)	0.638
Glucagon-like peptide-1, pg/mL	152.7 (103.8; 201.4)	190.7 (135.6; 289.0)	0.001
Glucagon, pg/mL	23.3 (14.1; 35.6)	22.7 (11.7; 35.9)	0.902
Interleukin-6, pg/mL	2.0 (0.3; 6.5)	1.7 (0.7; 3.7)	0.760
Insulin, pg/mL	584.5 (406.9; 831.9)	719.0 (584.5; 998.2)	0.082
Leptin, ng/mL	2.3 (1.2; 4.1)	6.2 (3.0; 1.2)	0.0001
Monocyte chemoattractant protein-1, pg/mL	127.5 (103.1; 1914.0)	138.7 (113.1; 201.9)	0.087
Pancreatic polypeptide, pg/mL	32.3 (21.8; 49.9)	35.1 (19.9; 52.5)	0.948
Polypeptide YY, pg/mL	51.3 (38.4; 74.3)	52.7 (36.0; 73.9)	0.753
Secretin, pg/mL	15.0 (6.8; 41.2)	13.2 (5.4; 37.6)	0.628
Tumour necrosis factor-α, pg/mL	2.7 (1.6; 4.0)	3.8 (2.4; 5.3)	0.003
Adiponectin, μg/mL	37.2 (26.8; 58.3)	29.9 (14.6; 40.3)	0.078
Adipsin, μg/mL	8.6 (5.8; 19.8)	7.6 (5.4; 15.4)	0.206
Lipocalin-2, ng/mL	409.9 (185.8; 657.7)	263.1 (154.6; 542.3)	0.095
Plasminogen activator inhibitor-1, ng/mL	19.7 (12.0; 33.7)	24.2 (14.0; 32.0)	0.296
Resistin, ng/mL	121.8 (63.0; 170.8)	118.1 (72.4; 169.3)	0.956

**Table 2:** Analysis of blood levels of adipokines/cytokines associated with the secretory activity of visceral adipocytes in men with hyper-LDL-C with and without AO (Me (25%; 75%)).

Parameters	LDL-C 10th Decile, AO (−) N = 40	LDL-C 10th Decile, AO (+) N = 47	*p*
Amylin, pg/mL	13.4 (8.0; 18.1)	14.7 (9.2; 21.9)	0.250
C-peptide, ng/mL	0.7 (0.5; 1.3)	1.4 (0.8; 1.8)	0.005
Ghrelin, pg/mL	54.3 (29.2; 120.7)	82.2 (29.2; 126.8)	0.649
Glucose-dependent insulinotropic polypeptide, pg/mL	28.8 (17.5; 45.1)	29.5 (19.6; 52.2)	0.514
Glucagon-like peptide-1, pg/mL	169.4 (109.9; 231.2)	223.1 (142.4; 334.1)	0.054
Glucagon, pg/mL	26.5 (15.8; 41.2)	26.4 (16.5; 43.4)	0.933
Interleukin-6, pg/mL	2.9 (0.5; 7.8)	1.5 (0.7; 3.6)	0.606
Insulin, pg/mL	584.5 (455.3; 926.2)	719.0 (584.5; 931.0)	0.486
Leptin, ng/mL	1.3 (0.9; 2.7)	3.6 (2.2; 6.8)	0.0001
Monocyte chemoattractant protein-1, pg/mL	144.7 (108.6; 205.5)	143.6 (21.8; 55.1)	0.544
Pancreatic polypeptide, pg/mL	36.7 (22.8; 49.5)	36.7 (21.8; 55.1)	0.859
Polypeptide YY, pg/mL	52.7 (40.8; 91.2)	59.1 (40.7; 83.3)	0.818
Secretin, pg/mL	11.5 (5.1; 41.6)	132 (5.7; 25.0)	0.956
Tumour necrosis factor-α, pg/mL	2.9 (2.1; 4.9)	3.3 (2.0; 5.4)	0.539
Adiponectin, μg/mL	38.5 (20.8; 67.0)	28.6 (13.5; 41.2)	0.177
Adipsin, μg/mL	10.6 (6.8; 25.4)	7.6 (5.3; 15.5)	0.126
Lipocalin-2, ng/mL	431.0 (254.6; 637.1)	257.9 (134.6; 545.7)	0.036
Plasminogen activator inhibitor-1, ng/mL	20.5 (13.2; 35.3)	23.4 (13.8; 34.5)	0.615
Resistin, ng/mL	110.6 (38.4; 171.9)	152.0 (85.0; 185.6)	0.273

**Table 3:** Analysis of blood levels of adipokines/cytokines associated with the secretory activity of visceral adipocytes in women with hyper-LDL-C with and without AO (Me (25%; 75%)).

Parameters	LDL-C 10th Decile, AO (−) N = 39	LDL-C 10th Decile, AO (+) N = 32	*p*
Amylin, pg/mL	8.6 (6.7; 13.6)	9.8 (7.1; 16.0)	0.323
C-peptide, ng/mL	0.7 (0.5; 0.8)	1.0 (0.7; 1.7)	0.003
Ghrelin, pg/mL	95.3 (29.2; 187.5)	66.0 (44.6; 1163.5)	0.485
Glucose-dependent insulinotropic polypeptide, pg/mL	23.5 (16.1; 34.3)	22.7 (13.5; 38.4)	0.764
Glucagon-like peptide-1, pg/mL	139.4 (92.3; 171.5)	162.4 (132.6; 257.0)	0.013
Glucagon, pg/mL	19.1 (10.7; 29.6)	14.9 (9.3; 32.9)	0.790
Interleukin-6, pg/mL	1.7 (0.2; 3.3)	2.0 (0.5; 3.7)	0.513
Insulin, pg/mL	406.9 (406.9; 803.7)	831.9 (451.3; 1179.4)	0.075
Leptin, ng/mL	3.7 (2.0; 6.2)	11.4 (8.1; 19.8)	0.0001
Monocyte chemoattractant protein-1, pg/mL	122.5 (92.3; 141.0)	132.4 (108.1; 189.8)	0.136
Pancreatic polypeptide, pg/mL	30.1 (19.9; 50.0)	33.9 (18.0; 45.3)	0.686
Polypeptide YY, pg/mL	48.2 (36.0; 67.4)	44.7 (36.0; 65.5)	0.549
Secretin, pg/mL	21.2 (8.6; 41.4)	17.8 (5.0; 65.1)	0.837
Tumour necrosis factor-α, pg/mL	2.2 (1.5; 3.7)	4.0 (2.7; 5.4)	0.0001
Adiponectin, μg/mL	35.2 (27.4; 58.0)	32.2 (16.4; 40.0)	0.247
Adipsin, μg/mL	7.8 (5.1; 16.1)	7.6 (5.6; 14.8)	0.862
Lipocalin-2, ng/mL	371.7 (152.5; 699.4)	329.8 (165.0; 561.1)	0.767
Plasminogen activator inhibitor-1, ng/mL	18.2 (11.6; 32.0)	24.5 (14.2; 29.9)	0.339
Resistin, ng/mL	131.5 (100.8; 169.6)	77.8 (57.1; 108.2)	0.064

**Table 4:** Results of a step-by-step regression analysis of the association of adipokines/cytokines with the presence of AO against a background of hyper-LDL-C in people aged 25–44 years.

Parameters	B	Exp B	95.0% C.I. for Exp B	*p*
Gender, M vs. F	1.590	0.204	0.080–0.517	0.001
Diastolic blood pressure	0.041	1.042	1.006–1.079	0.021
Triglycerides	0.784	1.080	0.588–1.984	0.001
Leptin	0.383	1.467	1.305–1.650	0.0001
Lipocalin-2	−0.001	0.999	0.997–1.001	0.086

**Table 5:** Results of a step-by-step regression analysis of the association of adipokines/cytokines with the presence of AO against a background of hyper-LDL-C in men aged 25–44 years.

Parameters	B	Exp B	95.0% C.I. for Exp B	*p*
Diastolic blood pressure	0.055	1.056	1.005–1.110	0.030
Leptin	0.788	2.198	1.555–3.108	0.0001

**Table 6:** Results of a step-by-step regression analysis of the association of adipokines/cytokines with the presence of AO against a background of hyper-LDL-C in women aged 25–44 years.

Parameters	B	Exp B	95.0% C.I. for Exp B	*p*
Diastolic blood pressure	0.054	1.056	1.002–1.112	0.041
Triglycerides	0.721	2.056	1.035–4.085	0.040
Leptin	0.307	1.359	1.201–1.538	0.0001
Tumor necrosis factor-α	0.400	1.491	1.070–2.078	0.018

## Data Availability

The datasets before and after analysis in this study are available from the corresponding author on reasonable request.
